# Supplementary material for: Exploring the Molecular Underpinnings of Cancer-Causing Oncohistone Mutants Using Yeast as a Model
Source: J Fungi (Basel). 2023 Dec 11;9(12):1187. doi: 10.3390/jof9121187 (PMC10744705; doi:10.3390/jof9121187)
Supplement: Supplementary file 1 [file jof-09-01187-s001.zip › jof-2673447-supplementary.pdf]

Supplemental Figure 1

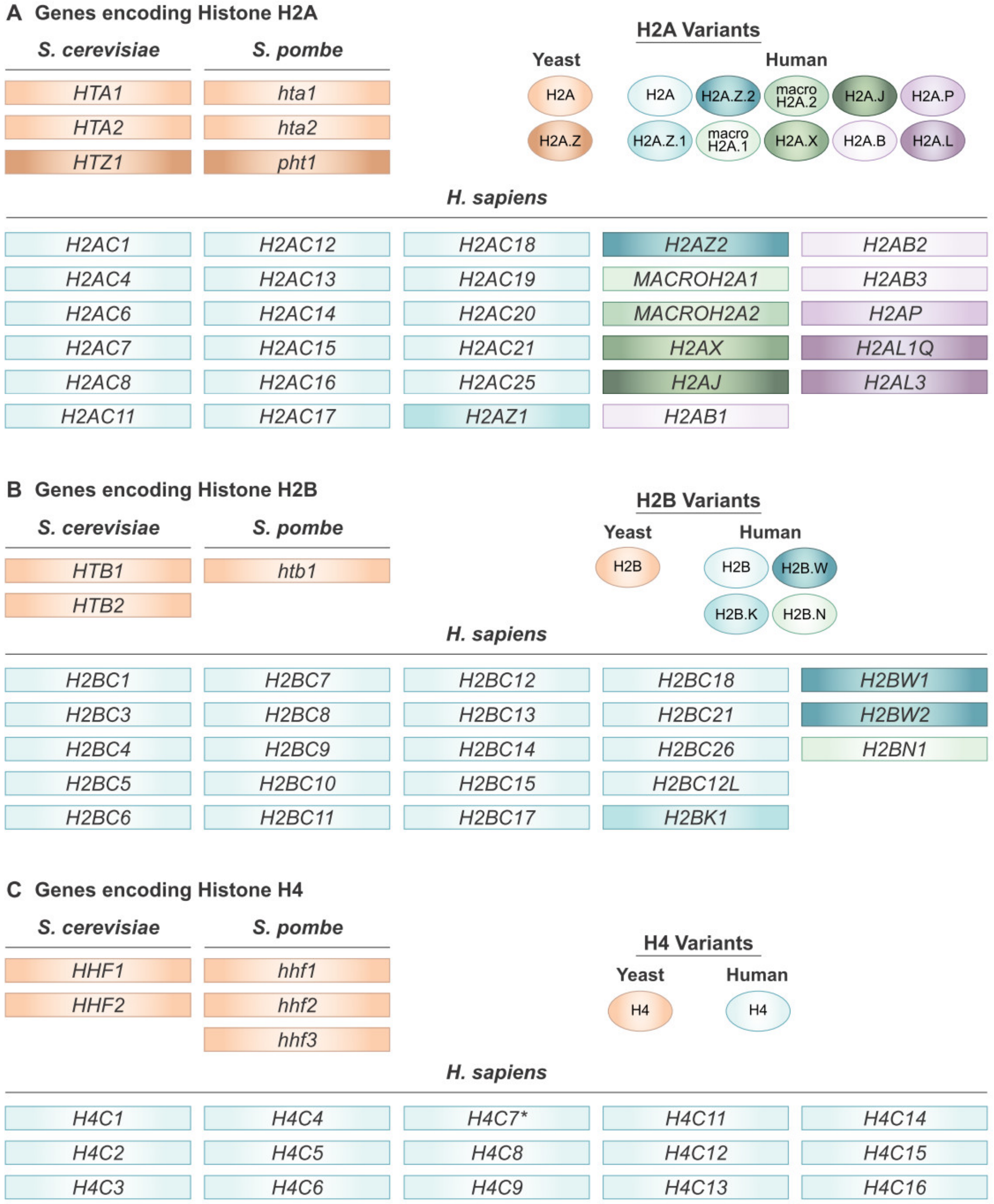

Figure S1: A comparison of histones H2A, H2B, and H4 from *S. cerevisiae*, *S. pombe*, and *H. sapiens*. Humans have many more gene copies and protein variants for **(A)** H2A, **(B)** H2B, or **(C)** H4 than either yeast species. The genes that encode each variant are color coordinated with their respective Histone Variants. The genes for H2A and H2B result in minor amino acid sequence variations for the canonical protein products which are not reflected here. *The existence of the protein encoded by this gene is uncertain, though there is evidence that it expresses the histone H4G variant .

Supplemental Figure 2

A H2A Protein Sequence Alignments

```
S.c. H2A    1 SGGKGGKAGSAAKASQSRSAKAGLTFPVGRVHRLLRRGNYAQRIGSGAPVYLTAVLEYLAAEILEL 66
H.s. H2A    1 SG-RGKQGGKARAKAKTRSSRAGLQFPVGRVHRLLRKGNYAERVGAGAPVYLAAVLEYLTAEILEL 65

S.c. H2A   67 AGNAARDNKKTRIIPRHLQLAIRNDDELNKLLGNVTIAQGGVLPPNIHQNLLPKKSAKATKASQEL 132
H.s. H2A   66 AGNAARDNKKTRIIPRHLQLAIRNDEELNKLLGKVTIAQGGVLPPNIQAVLLPKKTESHHKAKGK- 130
------------------------------------------------------------------
S.p. H2Aα   1 SG-GKSGGKAAVAKSAQSRSAKAGLAFPVGRVHRLLRKGNYAQRVGAGAPVYLAAVLEYLAAEILE 65
S.p. H2Aβ   1 SG-GKSGGKAAVAKSAQSRSAKAGLAFPVGRVHRLLRKGNYAQRVGAGAPVYLAAVLEYLAAEILE 65
H.s. H2A    1 SGRGKQGGKARA--KAKTRSSRAGLQFPVGRVHRLLRKGNYAERVGAGAPVYLAAVLEYLTAEILE 64

S.p. H2Aα  66 LAGNAARDNKKTRIIPRHLQLAIRNDEELNKLLGHVTIAQGGVVPNINAHLLPKTSGRTGKPSQEL 131
S.p. H2Aβ  66 LAGNAARDNKKTRIIPRHLQLAIRNDEELNKLLGHVTIAQGGVVPNINAHLLPKQSGK-GKPSQEL 130
H.s. H2A   65 LAGNAARDNKKTRIIPRHLQLAIRNDEELNKLLGKVTIAQGGVLPNIQAVLLPKKTESHHKAKGK- 129
```

	Sequence Identity with *S. cerevisiae* H2A
**H2A**	73%

	Sequence Identity with *S. pombe* H2A
**H2Aα**	78%
**H2Aβ**	78%

B H2B Protein Sequence Alignments

```
S.c. H2B    1 SAKAEKKPASKAPAEKKPAAKKTSTST--DGKKRSKARKETYSSYIYKVLKQTHPDTGISQKSMSI 64
H.s. H2B    1 -----PEPAKSAPAPKKGSKKAVTKAQKKDGKKRKRSRKESYSIYVYKVLKQVHPDTGISSKAMGI 61

S.c. H2B   65 LNSFVNDIFERIATEASKLAAYNKKSTISAREIQTAVRLILPGELAKHAVSEGTRAVTKYSSSTQA 130
H.s. H2B   62 MNSFVNDIFERIAGEASRLAHYNKRSTITSREIQTAVRLLLPGELAKHAVSEGTKAVTKYTSSK-- 125
------------------------------------------------------------------
S.p. H2B    1 -SAAEKKPASKAPAGKAPRDTMKSADKKRGKNRKETYSSYIYKVLKQVHPDTGISNQAMRILNSFV 65
H.s. H2B    1 PEPAKSAPAPKKGSKKAVTKAQKKDGKKRKRSRKESYSIYVYKVLKQVHPDTGISSKAMGIMNSFV 66

S.p. H2B   66 NDIFERIATEASKLAAYNKKSTISSREIQTAVRLILPGELAKHAVTEGTKSVTKYSSSAQ 125
H.s. H2B   67 NDIFERIAGEASRLAHYNKRSTITSREIQTAVRLLLPGELAKHAVSEGTKAVTKYTSSK- 125
```

	Sequence Identity with *S. cerevisiae* H2B
**H2B**	67%

	Sequence Identity with *S. pombe* H2B
**H2B**	69%

C H4 Protein Sequence Alignments

```
S.c. H4    1 SGRGKGGKGLGKGGAKRHRKILRDNIQGITKPAIRRLARRGGVKRISGLIYEEVRAVLKSFLESVI 66
H.s. H4    1 SGRGKGGKGLGKGGAKRHRKVLRDNIQGITKPAIRRLARRGGVKRISGLIYEETRGVLKVFLENVI 66

S.c. H4   67 RDSVTYTEHAKRKTVTSLDVVYALKRQGRTLYGFGG 102
H.s. H4   67 RDAVTYTEHAKRKTVTAMDVVYALKRQGRTLYGFGG 102
```

	Sequence Identity with *S. cerevisiae* H4
**H4**	92%

```
S.p. H4    1 SGRGKGGKGLGKGGAKRHRKILRDNIQGITKPAIRRLARRGGVKRISALVYEETRAVLKLFLENVI 66
H.s. H4    1 SGRGKGGKGLGKGGAKRHRKVLRDNIQGITKPAIRRLARRGGVKRISGLIYEETRGVLKVFLENVI 66

S.p. H4   67 RDAVTYTEHAKRKTVTSLDVVYSLKRQGRTIYGFGG 102
H.s. H4   67 RDAVTYTEHAKRKTVTAMDVVYALKRQGRTLYGFGG 102
```

	Sequence Identity with *S. pombe* H4
**H4**	91%

Figure S2: Protein alignment comparing histones H2A, H2B, and H4 sequences from *S. cerevisiae*, *S. pombe*, and *H. sapiens*. (A, B, C) The canonical protein sequences for each histone are compared between the *S. cerevisiae* or *S. pombe* protein and the human protein. Blue residues represent conservative changes, where the biochemical properties of the amino acid are maintained, and orange residues represent non conservative changes, where the biochemical properties are altered.
